# Ginsenosides Rb1 and Rg1 Stimulate Melanogenesis in Human Epidermal Melanocytes via PKA/CREB/MITF Signaling

**DOI:** 10.1155/2014/892073

**Published:** 2014-03-31

**Authors:** Mao Lin, Bao-Xiang Zhang, Ci Zhang, Nan Shen, Yun-Ying Zhang, Ao-Xue Wang, Cai-Xia Tu

**Affiliations:** ^1^Department of Dermatology, The Second Affiliated Hospital of Dalian Medical University, No. 467 Zhongshan Road, Dalian, Liaoning 116027, China; ^2^College of Integrative Medicine, Dalian Medical University, Dalian 116027, China

## Abstract

Reduced or defective melanin skin pigmentation may cause many hypopigmentation disorders and increase the risk of damage to the skin triggered by UV irradiation. Ginsenosides Rb1 and Rg1 have many molecular targets including the cAMP-response element-binding protein (CREB), which is involved in melanogenesis. This study aimed to investigate the effects of ginsenosides Rb1 and Rg1 on melanogenesis in human melanocytes and their related mechanisms. The effects of Rb1 and Rg1 on cell viability, tyrosinase activity, cellular melanin content and protein levels of tyrosinase, microphthalmia-associated transcription factor (MITF), and activation of CREB in melanocytes were assessed. Results showed that Rb1 or Rg1 significantly increased cellular melanin content and tyrosinase activity in a dose-dependent manner. By contrast, the cell viability of melanocytes remained unchanged. After exposure to Rb1 or Rg1, the protein levels of tyrosinase, MITF, and phosphorylated CREB were significantly increased. Furthermore, pretreatment with the selective PKA inhibitor H-89 significantly blocked the Rb1- or Rg1-induced increase of melanin content. These findings indicated that Rb1 and Rg1 increased melanogenesis and tyrosinase activity in human melanocytes, which was associated with activation of PKA/CREB/MITF signaling. The effects and mechanisms of Rb1 or Rg1 on skin pigmentation deserve further study.

## 1. Introduction

The content of melanin in the skin determines the darkness of skin color. Reduced melanin content or defective melanin metabolism may cause many hypopigmentation disorders and increase the risk of skin damage induced by UV irradiation. Many research groups are investigating the regulation of skin pigmentation with the goal of developing tanning cosmetics to reduce the risk of skin cancer and cure or prevent hypopigmentation diseases [1].

Melanin is synthesized in melanocytes via an enzymatic cascade with tyrosinase as a key enzyme. Enhanced tyrosinase activity may increase melanin production [2]. The microphthalmia-associated transcription factor (MITF) binds to the M-box within the tyrosinase promoter resulting in upregulation of tyrosinase gene expression [3–5]. The MITF is activated via the protein kinase A- (PKA-) cAMP responsive element binding protein (CREB) signaling pathway [4–6].

Ginseng, which is the root of* Panax ginseng*, has been used as a tonic remedy in traditional Chinese medicine for over 2000 years [7, 8]. Among more than 30 ginsenosides, Rb1 and Rg1 are regarded as the main active compounds responsible for many pharmaceutical actions of ginseng [9–11]. So far, most of the studies regarding Rb1 and Rg1 have focused on their impact on neural cells [12]. Studies have demonstrated that the actions of Rb1 and Rg1 are involved in the PKA/CREB signal transduction pathway. For example, Rb1 and Rg1 can activate PKA in rat cerebrocortical nerve terminals [13]. Ginsenoside Rb1 activates CREB in cortical neurons [14], and ginsenoside Rg1 enhances activation of the PKA/CREB pathway in cultured cortical neurons exposed to A*β* or glutamate-mediated synaptic stress [15]. In addition, in Schwann cells, the beneficial effects of Rb1 and Rg1 on proliferation and expression of nerve growth factor and brain-derived neurotrophic factor occur mainly through the PKA signaling pathway [13].

Melanocytes and neurons are both derived from the neural plate, and, more specifically, all neurons in the peripheral nervous system and melanocytes in the epidermis are derived from the neural crest. We hypothesize that ginsenosides Rb1 and Rg1 might also exert their influences on melanocytes via the PKA/CREB signaling pathway, which in turn regulates the expression of MITF and subsequently that of tyrosinase. However, whether Rb1 and Rg1 influence melanogenesis in human melanocytes is largely unknown. Therefore, the aim of the present study was to investigate the effects of ginsenosides Rb1 and Rg1 on melanogenesis in human melanocytes and their underlying molecular mechanisms, especially the mechanisms involved in PKA/CREB pathway.

## 2. Materials and Methods

### 2.1. Chemicals and Antibodies

Ginsenoside Rb1 (2-O-*β*-glucopyranosyl-(3*β*,12*β*)-20-[(6-O-*β*-D-glucopyranosyl-*β*-D-glucopyranosyl) oxy]-12-hydroxydammar-24-en-3-yl *β*-D-glucopyranoside, MW: 1109.29, purity ≥ 98% by HPLC), ginsenoside Rg1((3*β*,6*α*,12*β*)-3,12-dihydroxydammar-24-ene-6,20-diyl bis-*β*-D-glucopyranoside, MW: 801.01, purity ≥ 98% by HPLC), forskolin (FK), L-DOPA, methylthiazolyldiphenyl-tetrazolium bromide (MTT), dimethyl sulfoxide (DMSO), Triton X-100, PD98059, H-89, and Bradford reagent were all obtained from Sigma-Aldrich Chemical Co. (MO, USA). The antibodies recognizing phospho-CREB-1 (p-CREB-1, 10E9), total CREB-1 (t-CREB, H-74), tyrosinase (6A207), MITF (C5), and *β*-actin (I-19) were supplied by Santa Cruz Biotechnology Inc. (Santa Cruz, CA, USA).

### 2.2. Cell Cultures

Primary cultures of normal human melanocytes were established according to the method previously described with slight modifications [16]. Briefly, melanocytes were isolated from the foreskin of patients undergoing circumcision in our hospital and maintained in M254 medium supplemented with Human Melanocyte Growth Supplement (HMGS, Invitrogen, Carlsbad, CA, USA), 100 IU/mL penicillin, and 100 *μ*g/mL streptomycin (Invitrogen, Carlsbad, CA, USA). Cells were cultured in a humidified incubator in 5% CO_2_ at 37°C and used at passages 2–6. In this study, each experiment was conducted with melanocytes cultured from three different donors.

### 2.3. Measurement of Cell Viability

Cell viability was measured using a modification of the MTT assay reported by Im et al. [17]. Briefly, melanocytes were cultured in 96-well plates, 20 *μ*L of 5 mg/mL MTT reagent was added to each well with 180 *μ*L of culture medium, and the plate was incubated for 4 hrs. The media were then removed and the plates were shaken with DMSO for 5 min to dissolve the formazan crystals. The absorbance of the samples was measured at a wavelength of 490 nm.

### 2.4. Melanin Content Measurement

Melanin content was measured according to the method described by Oka et al. with a slight modification [18]. Briefly, the supernatant was discarded and cells were washed twice with PBS. Then 100 *μ*L of 1 M NaOH was added to each well at 80°C for 1 h in waterbath. Using microplate reader to measure melanin content at 400 nm, the melanin content was expressed by absorbance.

### 2.5. Assay of Tyrosinase Activity

Tyrosinase activity was estimated by measuring the rate of 3,4-dihydroxyphenylalanine (L-DOPA) oxidase activity as previously described [19]. Briefly, cells were solubilized with 1% Triton-100 and lysates were clarified by centrifugation at 10,000 g for 10 min. After protein quantification by the Bradford reagent method (Sigma-Aldrich) and protein levels were adjusted with lysis buffer, 80 *μ*L aliquots of each lysate (each containing the same amount of protein) were placed in the wells of a 96-well plate, with 20 *μ*L of 5 mM L-DOPA. After a 20 min incubation at 37°C, absorbance values were measured spectrophotometrically at 475 nm.

### 2.6. Western Blot Analysis

Western blot analysis was performed using a previously described method [19] with slight modifications. Briefly, whole-cell extracts were lysed in buffer containing 50 mM Tris (pH 7.4), 150 mM NaCl, 1 mM 4-(2-aminoethyl) benzenesulfonyl fluoride hydrochloride, 100 units/mL aprotinin, 10 mM NaF, 1 mM Na_3_VO_4_, and 1% Triton X-100. After protein quantification by the Bradford reagent method (Sigma-Aldrich) and adjustment of protein concentration with lysis buffer, equal amounts of 50 *μ*g of protein from each sample were loaded in the gel. Proteins in cell lysates were separated by sodium dodecyl sulfate-polyacrylamide gel electrophoresis (SDS-PAGE), transferred to polyvinylidene difluoride (PVDF) membranes, and then exposed to the appropriate antibodies. The blots were visualized by an enhanced chemiluminescence (ECL) system (Amersham Biosciences, NJ, USA) using horseradish peroxidase-conjugated anti-rabbit or anti-mouse secondary antibodies. The western blot assays are representative of at least three experiments.

### 2.7. Statistical Analysis

All data were expressed as mean ± SD. One-way analysis of variance (ANOVA) was used to compare mean values using the SPSS 11.0 software program for windows (SPSS, Inc., Chicago, IL, USA). An alpha value of *P* < 0.05 was considered statistically significant.

## 3. Results

### 3.1. Ginsenosides Rb1 and Rg1 Increased Melanin Content and Tyrosinase Activity in Melanocytes

**Figure 1:**
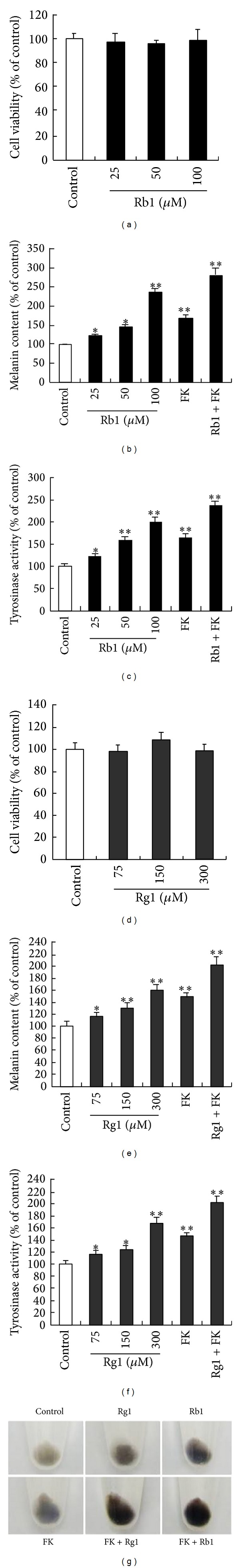
Ginsenosides Rb1 and Rg1 increased melanin content and tyrosinase activity in melanocytes. The cells were exposed to various concentrations of ginsenoside Rb1 or Rg1 or 10 *μ*M forskolin (FK, used as a positive control) or 10 *μ*M FK + 100 *μ*M Rb1 or 10 *μ*M FK + 300 *μ*M Rg1 for 72 hrs. Cell viability was determined by MTT assay ((a), (d)). The cellular melanin content ((b), (e)) and the cellular tyrosinase activity ((c), (f)) were shown as percentages of vehicle control. Cell pellets after treatment with 300 *μ*M Rg1, 100 *μ*M Rb1, 10 *μ*M FK, 10 *μ*M FK + 100 *μ*M Rb1, or 10 *μ*M FK + 300 *μ*M Rg1 were shown in (g). Data are expressed as the mean ± SD from three independent experiments that were carried out in triplicate. *P < 0.05, **P < 0.01, as compared with vehicle control (determined by one-way ANOVA). These experiments were conducted with melanocytes cultured from three different donors.

We first assessed the effects of ginsenosides Rb1 and Rg1 on cellular melanin content, tyrosinase activity, and cell viability. Forskolin is a chemical that was previously shown to promote melanogenesis via activation of CREB and upregulating expression of MITF and tyrosinase [6, 20] and was used as a positive control. As shown in Figures 1(a) and 1(d), cell viability was not changed significantly after 72 hrs treatment with 25–100 *μ*M ginsenoside Rb1 or 75–300 *μ*M ginsenoside Rg1. By contrast, melanin content and tyrosinase activity were increased in a dose-dependent manner (Figures 1(b), 1(c), 1(e), and 1(f)). Accordingly, the color of cell pellets was darker after being treated with Rg1 or Rb1 (Figure 1(g)). These results demonstrated that Rb1 and Rg1 promote tyrosinase activity and increase melanin content without influencing melanocyte viability.

### 3.2. Ginsenosides Rb1 and Rg1 Upregulated Expression of MITF and Tyrosinase

**Figure 2:**
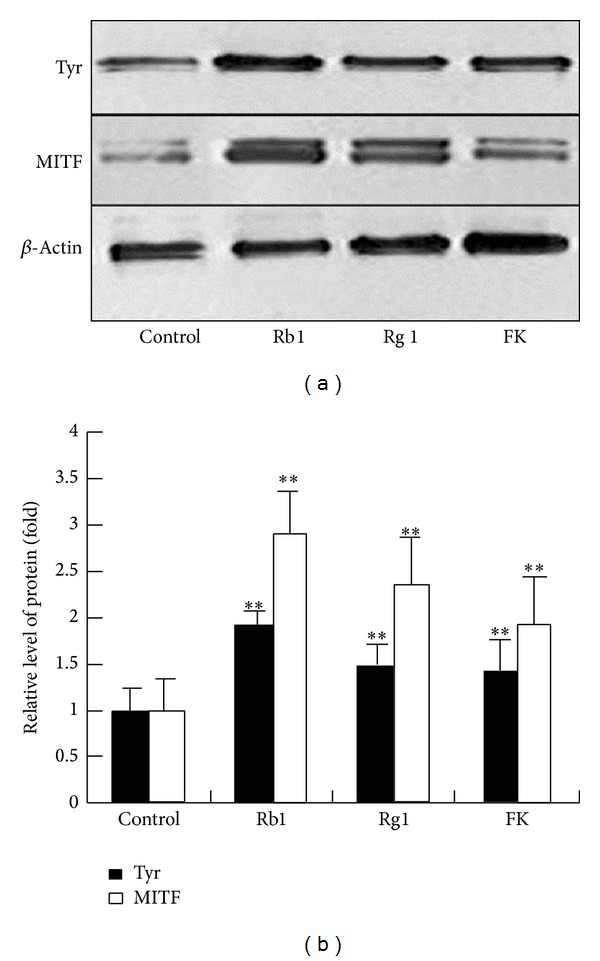
Ginsenosides Rb1 and Rg1 upregulated expression of MITF and tyrosinase. Cells were treated with Rb1 (100 *μ*M), Rg1 (300 *μ*M), or 10 *μ*M forskolin (FK, used as a positive control) for 24 hrs. The expression levels of MITF and tyrosinase (Tyr) proteins (a) were quantified and normalized to the level of *β*-actin by western blotting analysis. The normalized data for each was plotted as bar graphs (b). Data are expressed as the mean ± SD from three independent experiments carried out in triplicate. **P < 0.01, compared with the control (as determined by one-way ANOVA). These experiments were conducted with melanocytes cultured from three different donors.

To determine if the promelanogenic activity of Rb1 and Rg1 involves MITF, protein levels of tyrosinase and MITF in ginsenosides Rb1- or Rg1-treated melanocytes were determined by western blotting assay. As shown in Figure 2(a), the expression of MITF and tyrosinase in melanocytes was increased by Rb1 and Rg1, which suggested that the promelanogenic effect of Rb1 and Rg1 is associated with the upregulation of the MITF signaling pathway.

### 3.3. Ginsenosides Rb1 and Rg1 Triggered CREB Signaling Pathway in Melanocytes

**Figure 3:**
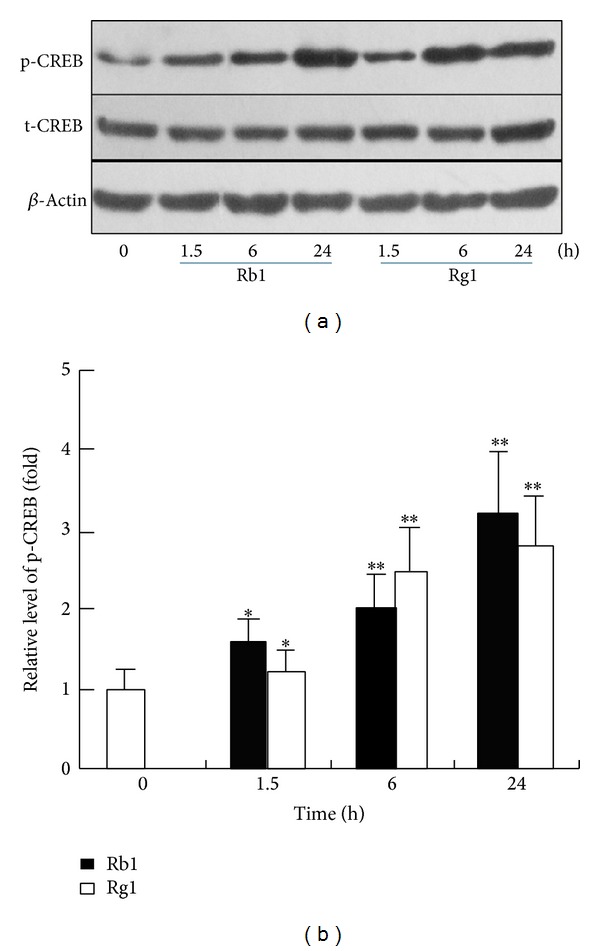
Ginsenosides Rb1 and Rg1 triggered CREB signaling in melanocytes. Melanocytes were treated with Rb1 (100 *μ*M), Rg1 (300 *μ*M) for the indicated times, and the levels of phosphorylated CREB (p-CREB) were quantified and normalized to the level of *β*-actin by western blotting analysis (a). The normalized data for each was plotted as bar graphs (b). Data are expressed as the mean ± SD from three independent experiments that were carried out in triplicate. *P < 0.05, **P < 0.01, as compared with the control (as determined by one-way ANOVA). These experiments were conducted with melanocytes that were cultured from three different donors.

To determine the effects of ginsenosides Rb1 and Rg1 on CREB signaling, melanocytes were treated with Rb1 or Rg1 for the indicated times, and western blotting analysis was performed. As shown in Figures 3(a) and 3(b), the expression levels of phosphorylated CREB were elevated at time points after 1.5 hrs. These results suggested that treatment with Rb1 or Rg1 induced activation of the CREB signaling pathway, which may be involved in the regulation of melanogenesis.

**Figure 4:**
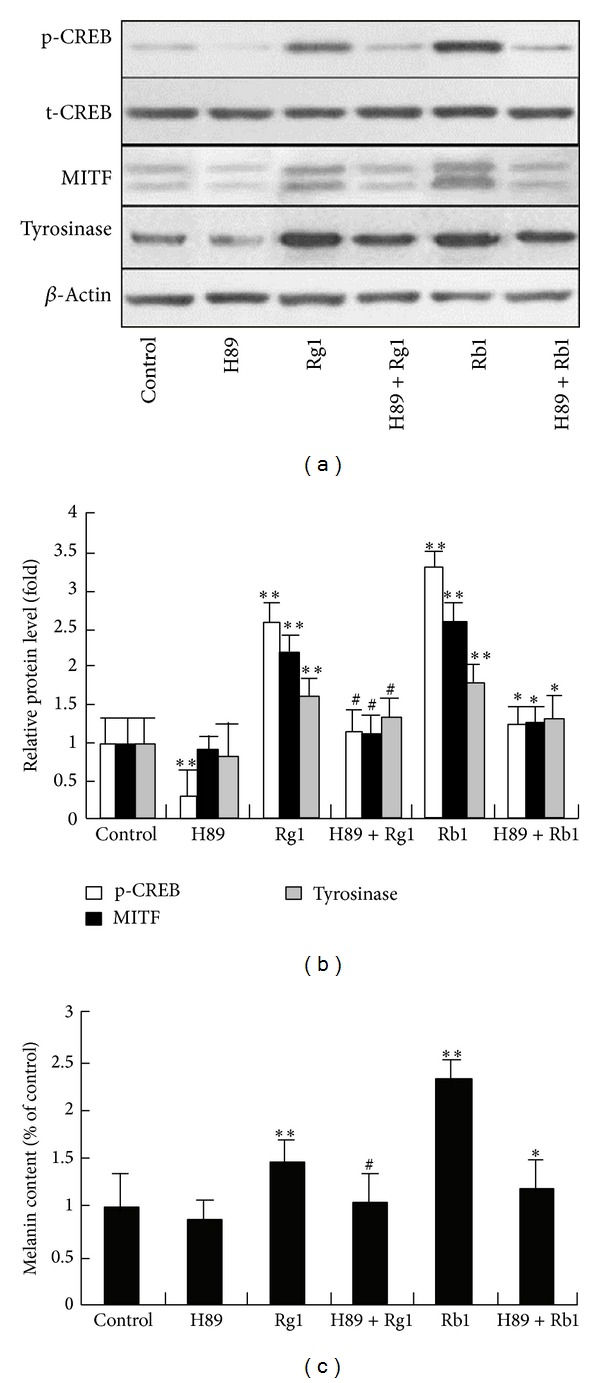
The effect of the PKA inhibitor on Rb1- and Rg1-induced MITF expression and melanin content in melanocytes. Melanocytes were exposed to ginsenoside Rb1 (100 *μ*M) or ginsenoside Rg1 (300 *μ*M) with or without pretreatment with the PKA inhibitor H-89 (10 *μ*M) for 1 hr. After a further 24 hrs of culture, the levels of p-CREB, MITF, and tyrosinase were quantified and normalized to the levels of *β*-actin by western blotting analysis ((a), (b)). After 72 hrs of exposure to Rb1 and Rg1, cellular melanin content (c) was determined. **P < 0.01, as compared with control; ^#^ *P* < 0.05, as compared with cells treated with ginsenoside Rg1 alone; *P < 0.05, as compared with cells treated with ginsenoside Rb1 alone (as determined by one-way ANOVA).

To further evaluate the role of CREB in Rb1- or Rg1-induced increase of melanogenesis, a selective inhibitor of PKA, referred to as H-89, was employed. Melanocytes were exposed to ginsenoside Rb1 (100 *μ*M) or Rg1 (300 *μ*M) with or without pretreatment with H-89 (10 *μ*M) for 1 hr. The results showed that H-89 pretreatment significantly inhibited Rb1- or Rg1-induced phosphorylation of CREB and expression of MITF and tyrosinase (Figures 4(a) and 4(b)) and significantly blocked the increased melanin content that was induced by ginsenoside Rb1 or Rg1 (Figure 4(c)). These results suggested that the promelanogenic activity of Rb1 or Rg1 significantly depended on activation of the PKA/CREB signaling pathway.

## 4. Discussion

Ginseng is one of the most widely used herbal medicines in human individuals. Nervous system diseases are the most widely investigated diseases among all others with respect to the therapeutic effects of ginseng and ginsenosides [21]. Both Rg1 and Rb1 are the representative constituents of ginseng. Many researchers believe that they share many beneficial effects of ginseng in the context of the therapy for nervous system diseases [8]. Melanocytes share common embryological origin, signaling molecules, receptors, and signaling pathways with cells of the nervous system [22]. Ginsenosides Rg1 and Rb1 might also modulate the function of melanocytes.

Melanogenesis is the main function of melanocytes. In the present study, we demonstrated that Rb1 and Rg1 increased the melanin content in cultured normal human melanocytes in a dose-dependent manner without cytotoxicity. Since Rb1 and Rg1 did not increase cell number, the possibility that the upsurges in melanin content may result from cell proliferation can be excluded. Additionally, 100 *μ*M Rb1 stimulated more melanin production than did 300 *μ*M Rg1, suggesting a stronger melanin-promoting ability of Rb1. In general, these results suggest that Rb1 and Rg1 could be useful for photoprotection and treating vitiligo.

In melanocytes, melanin synthesis is regulated by melanogenic enzymes, of which tyrosinase is a rate-limiting enzyme of melanogenesis [23]. MITF is a basic helix-loop-helix leucine zipper transcription factor that can transactivate the tyrosinase promoters and plays a central role in melanogenesis. Here, we showed that Rb1 and Rg1 upregulate both MITF and tyrosinase expression in melanocytes, resulting in an increase in melanin production. MITF is a key transcription factor for Rab27a [24], a protein that is important for melanosome transport and Pmel17 [25]. The expression of Pmel17 is required for melanosome matrix formation. Whether Rb1 and Rg1 can also promote melanosome biogenesis via upregulation of MITF and transport in the process of melanogenesis is unknown.

Previous studies have demonstrated that activation of PKA phosphorylates the transcription factor CREB, resulting in an induction of MITF expression [6, 26]. As mentioned above, MITF induces the expression of tyrosinase, which initiates the catalysis of melanin from tyrosine by the sequential hydroxylation. Our results showed that inhibition of PKA blocks Rb1- and Rg1-induced expression of CREB and MITF, as well as melanin production. These results strongly suggest that the PKA/CREB/MITF signaling pathway might play a crucial role in Rb1- or Rg1-induced melanogenesis.

In addition, ginsenosides Rb1 and Rg1 exerted melanin-regulating activity in the present study. However, their effects on another type of melanin-producing cell, melanoma cells, remain unknown. It is worth noticing that some natural plants, for instance,* Phytolacca decandra*, which contain saponins, may have anticancer potential against skin melanoma cells through activation of caspase-mediated signaling and reactive oxygen species (ROS) generation [27]. Ginsenosides are saponins, and it was previously reported that ginsenoside Rh2 induced apoptosis of melanoma cells [28]. Therefore, whether Rb1 or Rg1 has antimelanoma potential deserves further study.

Taken together, our results demonstrate that ginsenosides Rb1 and Rg1 promote melanogenesis in cultured human melanocytes. The PKA/CREB/MITF signaling pathway plays an important role in Rb1- or Rg1-induced melanogenesis. Additionally, Rb1 or Rg1 could be useful for skin photoprotection and may represent an alternative treatment for vitiligo.
